# Protocol for a cluster-randomised trial to determine the effects of advocacy actions on the salt content of processed foods

**DOI:** 10.1186/s12889-016-2743-4

**Published:** 2016-01-25

**Authors:** Helen Trevena, Anne Marie Thow, Elizabeth Dunford, Jason H. Y. Wu, Bruce Neal

**Affiliations:** 1 The George Institute for Global Health, Sydney Medical School, University of Sydney, PO Box M201 Missenden Road, Camperdown, Sydney, NSW 2050 Australia; 2 Menzies Centre for Health Policy, School of Public Health, University of Sydney, Charles Perkins Centre, Sydney, NSW 2006 Australia; 3 Carolina Population Center, University of North Carolina at Chapel Hill, Chapel Hill, NC 27516 USA; 4 The School of Public Health, Faculty of Medicine, Epidemiology and Biostatistics, Imperial College of Science, Technology and Medicine, Praed Street, Norfolk Place, London, W2 1 PG UK; 5 The Royal Prince Alfred Hospital, Sydney, NSW 2050 Australia

**Keywords:** Advocacy, Food companies, Salt reduction, Randomised trial

## Abstract

**Background:**

Corporate decisions affecting the composition of processed foods are a potent factor shaping the nutritional quality of the food supply. The addition of large quantities of salt to foods is incompatible with Australian Dietary Guidelines and the reformulation of processed foods to have less salt is a focus of non-governmental organisations (NGOs). There is evidence that advocacy can influence corporate behaviour but there are few data to define the effects of NGOs working in the food space. The aim of this study is to quantify the effects of advocacy delivered by a local NGO on the salt content of food products produced or marketed by companies in Australia.

**Methods/Design:**

This is a cluster-randomised controlled trial that will be done in Australia from 2013 to 2015 which includes 45 food companies. The 23 companies in the control group will receive no specific intervention whilst the 22 companies in the intervention group will receive an advocacy program based upon an established theory of change model. The primary outcome will be the mean change in sodium content (mg/100 g) of processed foods produced or marketed by intervention compared to control companies assessed at 24 months. Interim outcomes (statements of support, published nutrition policies, level of engagement, knowledge and use of technology to reduce salt, salt reduction plans, and support for national initiatives) will also be assessed and a qualitative evaluation will provide more detailed insight.

**Discussion:**

This novel study will provide robust randomised evidence about the effects of advocacy on food company behaviour and the quality of the processed food supply. A finding of improved food company behaviour will highlight the potential for greater investment in advocacy whilst the opposite result will reinforce the importance of government-led initiatives for the improvement of the food supply.

**Trial registration:**

ClinicalTrials.gov: NCT02373423. 26/02/2015

**Electronic supplementary material:**

The online version of this article (doi:10.1186/s12889-016-2743-4) contains supplementary material, which is available to authorized users.

## Background

The food system, which includes the actors, institutions and processes that influence the way in which food products are produced, processed and distributed to consumers, is dynamic and complex [1–3]. Advances in food processing, defined as the use of a “series of mechanical or chemical operations to change or preserve food” [4], have enabled the launch and renovation of many food products targeted to meet consumer demand for safe, palatable, and convenient food. Likewise, distribution channels and retail operations have evolved to ensure processed foods are readily available to purchase. Decisions about the nutritional quality of processed foods and the numbers of foods marketed are taken primarily by food companies to create and respond to consumer demand and are potent factors shaping the food supply, and thereby food choice [4–9]. From a public health nutrition perspective, product formulations requiring the excess addition of salt, sugar, and saturated fat during processing are at odds with the Australian Dietary Guidelines which advise people to limit their intake of foods containing salt, sugar and saturated fat [10].

Salt reduction has been a recent focus of efforts to improve the quality of the food supply in a number of countries around the world [11]. At 8–10 g/day [12, 13], current salt intake in Australia is about double the World Health Organization (WHO) maximum of 5 g/day [14] and the Australian government suggested dietary target of 4 g/day [15]. In all likelihood excess dietary salt is causing large numbers of Australians to suffer from high blood pressure which causes premature stroke, heart attack and kidney disease – the leading causes of death and health care expenditure in the country [16]. While the results of occasional studies continue to produce debate about the effects of salt on health [17], systematic overviews that summarise the totality of the available data are indicative of harm [18, 19].

In a typical western diet about 75 % of salt derives from processed foods [20] with the rest being naturally occurring sodium in fresh products or added during food preparation or at the table. Many processed foods have a high salt content and there are often wide variations in the content of directly comparable products, despite the wealth of evidence about the harmful effects of excess salt on health [21–23].

An extensive body of literature presents different perspectives on how companies respond to macro-environmental and industry sector factors in terms of their organisational structure and size, culture, leadership, corporate learning and their approach to strategy development [1, 24–29]. Far less is known about the effect of advocacy on corporate actions and the literature describing the impact of public health advocacy is particularly sparse. The fast moving nature of the business environment and the messy nature of advocacy make quantification of effects especially challenging [30–32]. While there is some evidence that public health advocacy does have the potential to influence corporate behaviour [33–35] few robust data exist to describe the effects of advocacy programs on food companies [31, 36]. Accordingly, the goal of this study is to quantify the effects of public health advocacy delivered by an Australian non-governmental organisation (NGO) on the salt content of food products produced or marketed by companies in Australia.

## Methods

**Fig. 1:**
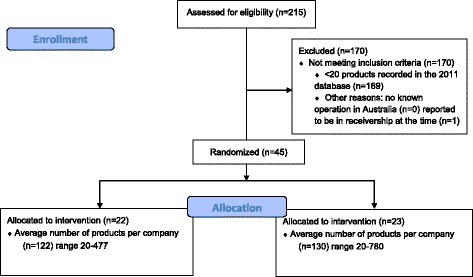
Flowchart: the progress of food company clusters and average cluster size used in randomisation

This study is a cluster-randomised controlled trial in which food companies are the unit of randomisation and average salt content of the products produced by the companies is the primary outcome (Fig. 1). The study is being done in Australia and was commenced in December 2013 with a scheduled completion date of December 2015. Ethics committee approval was obtained from the University of Sydney Human Research Ethics Committee for the conduct of interviews used to collect information about the food companies that was not already in the public domain with written informed consent obtained from all survey participants.

### Companies included

The food manufacturing sector includes industries involved in the preparation of processed, packaged, and shelf-ready foods as seen in supermarkets and the ingredients that go into the manufacture of dairy, cereal, baked goods, meat and fish, oil and fat, and processed fruit and vegetable products. In Australia in 2013–14 the Australian Food and Grocery Council reported there were 5,356 food manufacturing companies falling under this description with the main sectors described as meat and meat processing (21 %), bakery (15 %), dairy (8 %) and ‘other processing’ (41 %)—processing not elsewhere classified such as frozen pre-prepared meals, seasonings [37].

We used an established branded food composition database to select food companies for the study. The database [38] can be used to measure changes in food composition and includes an annual representative sample of foods for sale in leading Australian supermarkets. Companies were included in our study if they had Australian-based production, distribution or marketing operations of processed foods, and had 20 or more processed food items recorded in 2011. They were also required to be active in a sector involving some foods that were processed and contained added salt, sugar or saturated fat (Table 1).

**Table 1:** Inclusion and exclusion criteria for food companies and food groups

	Inclusion criteria	Exclusion criteria
*Food companies*	20 or more food products recorded in the database (2011).	Less than 20 food products recorded in the database (2011).
	Operations (production, distribution, marketing) in Australia.	Importers with no identifiable contact in Australia.
		Food companies in the database known to be in receivership^a^
*Major food groups and products*	Food products likely to contain added salt, sugar, or saturated fat:	Food products unlikely to contain added salt:
	Bread and bakery	Eggs
	Cereal and cereal products	Confectionery
	Convenience foods	Non-alcoholic beverages
	Dairy	Special foods^b^
	Edible oils and emulsions	Sugars, honey and related products
	Fish and fish products	Vitamins and supplements
	Meat and meat products	
	Sauces and spreads	
	Snack foods	
	Fruit and vegetables	

^a^Where a third party is appointed to take responsibility for the company assets. This may occur in a situation where a company cannot meet its financial responsibilities (for instance, insolvency)

^b^ Includes baby food, meal replacements, fitness and diet products, breakfast beverages, and sport protein powders

Australian Bureau of Statistics (ABS) definitions [39] were used to categorise companies as large (200+ employees), medium (20–199 employees) or small (0–19). Company ownership was determined using publicly available information and defined as being public or private [40]. The Australian New Zealand Standard Industrial Classification 2006 (ANZSIC) [41] was used to place companies into one or more food processing industry sectors. ANZSIC codes are used for the collection and reporting of financial, administrative and statistical data by government as well as by academics and the private sector [42]. The major food product categories in The George Institute Branded Food Composition Database were mapped against the ANZSIC system to identify the appropriate ANZSIC codes for each company. We identified 16 ANZSIC codes covering the relevant food manufacturing, grocery retailing and distribution (wholesaler) sectors (Table 2).

**Table 2:** Baseline characteristics of intervention and control groups showing industry sector presence and company characteristics

Baseline characteristics of companies	Number	Intervention	Control
		n (%)	n (%)
Food companies:	45	22	23
Participation in industry sector^a^			
Cereal, Pasta and Baking Mix Manufacturing	17	9 (41)	8 (35)
Other Food Product Manufacturing n.e.c	24	14 (64)	10 (43)
Seafood Processing	11	7 (32)	4 (17)
Fruit and Vegetable Processing	25	13 (59)	12 (52)
Meat and Meat Product Manufacturing	1	1 (5)	0 (0)
Cured Meat and Smallgoods Manufacturing	7	3 (14)	4 (17)
Ice Cream Manufacturing	6	2 (9)	4 (17)
Cheese and Other Dairy Manufacturing	13	6 (27)	7 (30)
Milk and Cream Processing	8	3 (14)	5 (22)
Oil and Fat Manufacturing	5	2 (9)	3 (13)
Biscuit Manufacturing (Factory-based)	11	6 (27)	5 (22)
Bread Manufacturing (Factory-based)	8	4 (18)	4 (17)
Confectionery Manufacturing	6	2 (9)	4 (17)
Cake and Pastry Manufacturing (Factory-based)	5	2 (9)	3 (13)
Potato, Corn and Other Crisp Manufacturing	6	3 (14)	3 (13)
Meat Processing	4	2 (9)	2 (9)
Company size			
Large (≥200 employees)	36	17 (77)	19 (83)
Small-medium (<200 employees)	9	5 (23)	4 (17)
Company ownership			
Private	19	8 (36)	11 (48)
Public	26	14 (64)	12 (52)
Commitment to salt reduction 2010–2012 (Australian Division of World Action on Salt and Health)	10	4 (18)	6 (26)
Participant of any Food and Health Dialogue salt reduction target 2009-2013	22	9 (41)	13 (57)
Use of Heart Foundation Tick logo on front-of-pack 2013	24	9 (41)	15 (65)
Member of Australian Food and Grocery Council Healthy Commitment 2013	7	3 (14)	4 (17)

^*a*^Australian and New Zealand Standard Industrial Classification (ANZSIC) [42]. One or more ANZSIC class descriptions can apply to a single food company

### Intervention

**Fig. 2:**
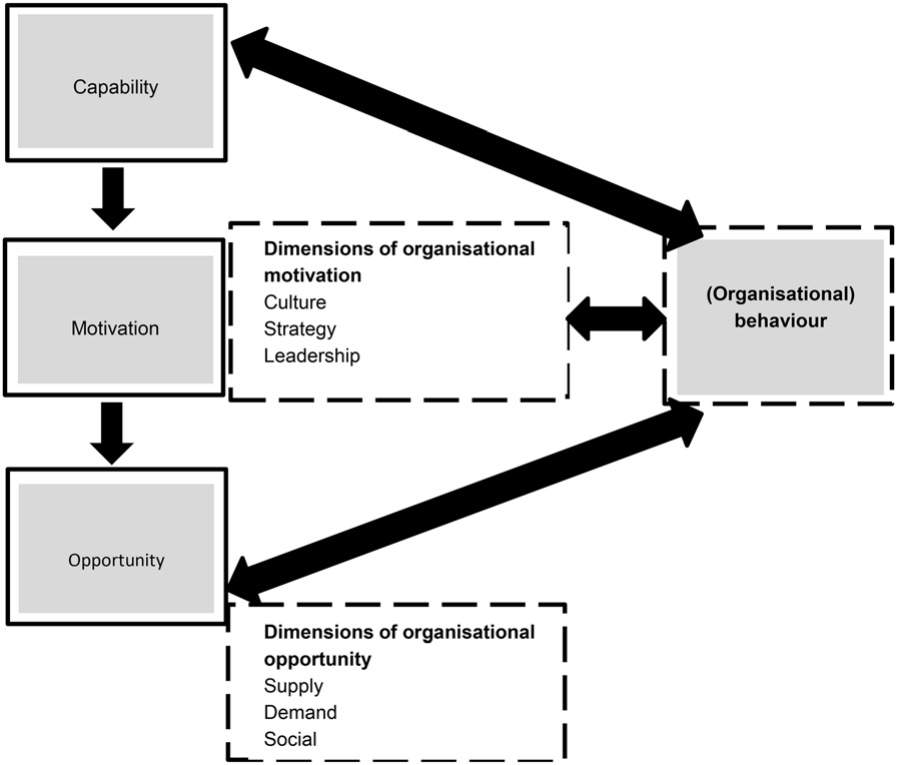
Change model: the original behavioural model by Michie et al. [43] of capability, motivation, opportunity and behaviour (COMB-B) is shown in grey. The organisational dimensions in the context of a healthier processed food supply are shown in the boxes with a dotted line

The intervention program will span 24 months from 2013 to 2015 and will be delivered on top of ongoing background activities seeking to improve the healthiness of food products as part of other governmental and non-governmental initiatives. The trial intervention program comprises of a series of advocacy actions based upon an established theory of change model (COM-B). The model defines three aspects of behaviour (capability, opportunity, and motivation) and maps these to intervention functions targeted to change one or more aspects of behaviour [43]. Drawing on organisational behavioural theory we have adapted the original definitions of capability, opportunity, and motivation [43] to an organisational context. Figure 2 depicts our adaption of the COM-B model to target organisational behavioural dimensions with a series of advocacy interventions designed to deliver a healthier processed food supply. The intervention program logic model (See Additional file 1) illustrates the overall design of the program and the connections between the theory of change model and the intended advocacy outcomes. A series of inputs have been identified indicating the resources and competencies required for the program. These inputs enable nine intervention functions [43] which are akin to ‘tools’ or’methods’ (training, coercion, incentivisation, persuasion, education, restriction, environmental restructuring, modelling) and these in turn enable the advocacy actions. The advocacy actions listed are those commonly used in advocacy programs of diverse types [36]. Collectively, the resources, intervention function, and advocacy actions represent the advocacy output, or, the ‘what we will do’ aspects of the program. Downstream from the advocacy outputs are the planned advocacy outcomes divided into interim (0–18 months) and long-term (18 months onward) outcomes. The interim outcomes are grouped by the change in organisational behaviour being targeted (opportunity, motivation, capability) with an advocacy action. During the intervention a feedback loop will enable monitoring of the intervention and interim data will be used to inform future actions. Finally, each advocacy action is targeted towards individuals in food companies who are considered to be influential representatives within their organisation, and in a position to favorably modify the national corporate agenda in their capacity as an ‘internal advocate’ of salt reduction.

### Control

Food companies assigned to the control group will have no specific intervention targeted at them as part of this study. The control group companies will, however, be exposed to ongoing background activities seeking to improve the healthfulness of food products across multiple sectors implemented as part of other governmental and non-governmental initiatives. In addition, if control group food companies make specific requests of the study team, such as for meetings or advice, these requests will be acted upon as far as is possible within the resources available.

### Randomisation

Company ownership, size, and industry sector [28, 44–46] may influence corporate motivation and capability to produce and market more healthful processed food products. Baseline data for eligible food companies were collected and companies classified into three strata based on company ownership, size of company and industry sector. 18 blocks were created with companies in each block randomised in a 1:1 ratio to intervention or control. Randomisation was performed in December 2012 by an independent statistician using a random number function in SAS (Version 9.3). Food companies were not made aware that they had been randomised to intervention or control as a part of this study. The only individuals aware of the randomised allocations were those directly associated with the research team implementing the intervention program.

### Data collection

Data for this study will derive from periodic surveys of the characteristics of included companies, annual surveys of the composition of the processed foods they provide and a log recording all elements of the intervention program implemented.

#### Surveys of companies

The collection of company data will be carried out at baseline, halfway through the intervention period and at study completion. The survey comprises a structured questionnaire focusing on the business context, company policies and practices related to product innovation and nutrition. The questionnaire is accompanied by an in-depth interview designed to seek a richer understanding of the Australian political and economic environment, stakeholder influence, and the development of company strategy in relation to producing and marketing healthier processed foods, an important component of which are company research and development (R&D) plans, and provides an opportunity to clarify queries the participants have about the survey questionnaire. The in-depth interviews done after baseline will provide an opportunity to probe for examples of NGO influence as a direct result of implementation of the advocacy program. The data collected will enable the full assessment of each aspect of the advocacy program depicted in the logic model. Interviews are sought with the most senior individual able to provide the requisite knowledge as nominated by the CEO or equivalent. The multiple roles of managers [1] suggest this approach will provide the breadth of data sought and that these data are likely to be representative of a food company’s approach to nutrition. In addition, relevant information available from public sources will be systematically collected and compiled for all companies. For example, data about engagement with other initiatives designed to improve the healthiness of processed foods, such as national and international affiliations, trade association-led healthy eating schemes [47], the Australian Food and Health Dialogue [48], the National Heart Foundation Tick [49], and the Australian Division of World Action on Salt and Health [50]. Knowledge about engagement with other campaigns to improve the healthiness of products is particularly important since it is likely to be influential in creating a more favourable climate for the advocacy work done for this project [34]. A short survey questionnaire will be used to collect data as part of an advocacy action(s) to assess knowledge and interest in salt reduction, knowledge of how to reduce salt, support and use of salt replacers/technology.

#### Nutritional data

The nutritional composition of the foods produced by the companies is recorded each year as part of an ongoing monitoring program [38]. For this project baseline data were collected between September and December 2013 and there will be annual collection of the data 12 and 24 months later. Data are obtained directly by taking a set of photographs of each product using a smartphone app designed to collect photos of packaged food items. One of the photographs includes the mandatory Nutrition Information Panel (NIP) and is available for a sample of about 20,000 Australian packaged food products each year. The included products are those on the shelves of the same four supermarkets (comprising a Coles, Woolworths, ALDI and IGA outlet) in Sydney, Australia. Where the same product is for sale in more than one supermarket it is recorded only once and likewise, where the same product is presented in different pack sizes, only one entry is recorded. For each food product the manufacturer, brand and product name, as well as the full nutrient profile on the NIP is recorded. These data are entered according to standardized procedures [51] and verified according to a defined quality assurance protocol and workflow, which includes screening for outliers and missing values, checking data entry accuracy and resolving queries and discrepancies by reference to the original NIP data, consultation between the research personnel, review of the manufacturer website or follow-up with the manufacturer directly.

#### Intervention monitoring data

Using an established approach [52, 53] and following the design set out in the program logic model, quantitative and qualitative data will be gathered for each advocacy action using advocacy logs (developed in Microsoft Word/Excel). The purpose of the advocacy log is to record the extent to which the interim advocacy outcomes as shown in the program logic model and further described (See Additional file 2) have been implemented as planned to achieve the desired change. The logs will be used to document the hypothesised theory of change underpinning each advocacy action; macro factors assumed to be influential; measures of the interim outcome; and progress in meeting them. Unintended consequence will also be documented. The types and sources of data that are anticipated to be collected are shown and will be recorded for both intervention and control companies.

### Study outcomes

#### Primary outcome

The primary outcome will be the mean sodium content of processed packaged foods produced and marketed by intervention compared to control companies reported in mg/100 g. For the primary outcome, ten major food categories will be included based on their known contribution to sodium in the diet (Table 1). Primary analyses will be made for all products combined but subsidiary analyses will explore the effects in each major product category and subsets of product categories that have and have not been targeted by the Food and Health Dialogue [48]. The primary examination of these outcomes will be after at least 18 months of intervention has been implemented using data collected in Q4 of 2015.

#### Interim outcomes

The interim outcomes (See Additional file 2) are listed below.

- The number of and type of publicly-available statements from food companies expressing support/non-support for healthier processed foods.
- The number of food companies with a nutrition policy published on their website.
- The level of engagement with the non-governmental organisation.
- The number of companies supporting the use of salt replacers/technologies in food processing to reduce the quantity of sodium required in processing.
- The number of companies supporting national salt reduction initiatives.
- The number of companies providing evidence of planned salt reduction.

#### Exploratory outcomes

Exploratory outcomes will be changes in average saturated fat (g/100 g), sugars (g/100 g), and energy density (kJ/100 g).

#### Sample size

The sample size for the study is 45 food companies and this will provide 80 % power to detect a difference of 50 mg/100 g in mean sodium content between the groups assuming the mean sodium content of products is 430 mg/100 g, the standard deviation for the sodium concentration is 300 mg/100 g and the intra-cluster correlation between products produced by the same company is 0.05 using a two-sided *T*-test with a significance level of 0.05. This would constitute an approximate 12 % reduction in sodium content of the foods produced by the intervention companies compared to the foods produced by the control group companies.

### Data analysis

#### Primary outcome - quantitative

The primary null hypothesis to be tested is that there is no difference in the change in mean sodium content of foods from baseline to follow-up between intervention and control group companies. Comparable null hypotheses will be tested for each of the secondary and exploratory outcomes related to organisational actions and other measures of food composition. The characteristics of the companies and their food products will be summarised with continuous metrics reported as means with standard deviations (or medians with ranges if data are substantially skewed) and categorical characteristics as proportions.

The effects of the intervention on the primary outcome and other continuous variables will be determined using linear mixed models. Effects on categorical outcomes will be made using non-linear mixed models. All analyses will be done on the intention-to-treat basis with methods including adjustment for intra-cluster correlation. A p-value <0.05 will be deemed to indicate statistical significance. Primary analyses will be uni-variable but if there is evidence of imbalance in baseline characteristics between intervention and control companies’ adjusted models will be fitted to explore the likely impact of the imbalances. Quantitative statistical analyses will be done using Stata 13.1 (Stata Corp, College Station, TX, USA) and SAS (Version 9.3).

#### Interim outcomes

##### Quantitative

The number and proportion of food companies with the variable of interest will be calculated using Pearsons’s chi-squared test or Fisher’s exact test to assess for differences between control and intervention.

##### Qualitative

An impact pathway approach [52] will be adopted to monitor and evaluate the predicted advocacy outcomes, as underpinned by a theory of change, with actual outcomes. Qualitative data will be used to identify and understand the impact of advocacy actions on food company behaviour and the potential impact of influences other than advocacy will be taken into account to identify alternative hypotheses as to why an advocacy outcome may have arisen. For example events in the macro environment such as a policy change in food labelling. There will be a summary for each advocacy action, which will include the relevance of the theory of change to the outcome, an assessment of the quality of the implementation (i.e. frequency and intensity of the intervention, appropriateness of targeting and impact on output indicator) and lessons learned to inform the subsequent advocacy actions [53]. The interim qualitative assessments are expected to yield information on the organisational opportunity, motivation and capability of food companies to reduce salt across the product portfolio and a richer understanding of the role of advocacy actions in effecting such a change.

The data from the in-depth interviews are anticipated to inform the assessment of the advocacy program. Interview data will be open-coded and organised using NVivo software (version 10; QSR International, Doncaster, Victoria, Australia). A framing matrix will be developed to organise, identify and systematically code data in an iterative process. The data will be subsequently organised and analysed into key themes developed deductively and inductively to identify patterns and interpret meaning.

## Project status

Ethics approval to collect survey questionnaire and interview data from food companies has been obtained from the Human Research Ethics Committee at the University of Sydney in Australia. There were 45 food companies selected for the study and between them they supplied 80 % of the products in the 2011 database with their products encompassing all the major product food groups for sale in Australian supermarkets. All included companies were invited to participate in the baseline survey conducted between December 2013 and April 2014. These data have informed the design of the advocacy program that is currently being implemented. Baseline nutritional data (2013) were collected in the 4th quarter of 2013. Final evaluation of the primary outcome using 2015 nutritional data will occur in 2016 and an interim evaluation will be conducted at the end of 2015.

## Discussion

This project will use a robust clustered randomised design to provide high quality evidence about the potential for an advocacy program to influence corporate behaviour and the healthiness of the processed food supply in Australia. With evidence of this type very limited, it is anticipated that the study findings will receive significant interest and widespread dissemination. A finding of improved company behaviour will provide new impetus for the many advocacy groups working in this space and the opposite result will serve to highlight the importance of government action for the enactment of change to the processed food supply. Furthermore, while the focus of this work is on the processed food supply, the novel experimental approach taken here will likely engender interest across a broader range of public health activities where there is a desire to know more about the effectiveness of advocacy.

The advocacy program design incorporates an adapted theory of change and intervention functions targeting organisational behaviour. Our conceptualisation of COM-B draws on the organisational behavioural literature as we considered it applicable to food companies seeking to produce and market healthier processed foods in Australia. As such, *organisational capability* is viewed as the technical, managerial, and process competencies necessary to improve the nutritional profile of processed foods [54]. In the same way, *organisational opportunity* comprises the physical supply and demand considerations where ‘supply’ is a set of up-stream activities (procurement and the availability and access to less salty ingredients) and ‘demand’ a set of down-stream activities (distribution channels, consumer preferences). Organisational opportunity also includes social opportunity [43] defined as the opportunity to contribute to the prevailing discourse and determined by organisational culture [55] (communications strategy, and framing). Lastly, *organisational motivation* is considered to be the mix of planned and unplanned events prompting an organisation to choose certain behaviours which may be planned positions on nutrition integral to the company culture or emergent positions responding to a threat to reputation or other issues [45].

Key features of the approaches to advocacy identified and applied in program development were the framing of the issue [56], the use of surveillance, the reporting of market-based activities, and holding companies to account – both praising good practice and identifying when companies fail to achieve benchmarks [36]. The study design will help to identify under what circumstances corporate actions to improve the food supply can be encouraged and identify the windows of opportunity for advocacy.

A key challenge in working with food companies is the risk of non-participation [57] and thereby the absence of opportunity to influence. Lack of trust and commercial sensitivities (for example disclosure of R&D plans) can be key barriers in this regard. Ongoing monitoring of the implementation, which will include an assessment of the opportunity/capacity to influence, and questions about the frequency, intensity, and reach of the advocacy actions will help us to explore this issue and seek solutions to it [58].

This study is highly novel in the use of a randomised design although the relatively small sample size will provide power to detect only moderately large effects of the advocacy program on the primary outcome. The measurement of advocacy and attribution of impact will also be difficult because of the complex, multifaceted nature of the intervention program [32, 36] and the shifting background political, economic and social contexts [30, 36]. Randomisation should help to control the latter issue since background environmental changes will influence both intervention and control groups but it is possible that there will be interactions between parts of the intervention and the environment which will be hard to identify. The concurrent qualitative evaluation of the program which includes the recording of notable macro-environmental factors will, however, provide some evaluation of these types of influences and add rigor and depth to the analysis of the overall study findings. Our application of an established plan for advocacy as depicted by our logic model could be a limitation as it has the potential to impair program effectiveness [32, 36] because it imposes a degree of rigidity to the program and may limit the capacity to respond to evolving opportunities. On the other hand the a priori definition of a change model and a series of core activities will aid replication of the intervention in other settings if it is proved effective. We acknowledge that the study evaluation will focus on the short- and medium-term effects and corporate change may take longer than our study period allows for. However, as part of an ongoing monitoring program we will be able to assess changes in mean salt content post-2016. Lastly, market share data for individual products are unavailable to us and are unlikely to become available as the cost is prohibitive, as well as these data generally coming with restrictions on their use. Weighting of the analyses by sales will not therefore be possible. The systematic collection of data from all products on shelves does however provide for subset comparisons of baseline products available in multiple years and new market introductions.

In conclusion, this study will provide evidence about the potential for an advocacy program to influence corporate behaviour and the healthiness of the processed food supply in Australia. Whether the program is effective or not, the trial results, which use a novel and highly robust design, will have important implications for the future of Australian efforts to reduce the large burden of disease caused by poor diet - a finding of improved company behaviour will highlight the need for investment in advocacy whilst the opposite finding will reinforce the importance of other policy-based initiatives for the improvement of the healthiness of the food supply.

## Additional files

Additional files 1: **Intervention progam logic model.**

Additional files 2: **Interim outcomes: examples of the types and sources of data, and measures.** Description of data: description of the interim measures, examples of the types of data to be collected, the data sources, and the measure. (PDF 175 kb)

## Abbreviations

NGO: Non-governmental organisations
ANZSIC: The Australian New Zealand Standard Industrial Classification 2006 (Revision 1.0)
COM-B: Capability, opportunity, motivation – behaviour
NIP: Nutrition Information Panels
WHO: World Health Organization
