# Supplementary figures and images for: Protocol for a cluster-randomised trial to determine the effects of advocacy actions on the salt content of processed foods
Source: BMC Public Health. 2016 Jan 25;16:75. doi: 10.1186/s12889-016-2743-4 (PMC4727283; doi:10.1186/s12889-016-2743-4)

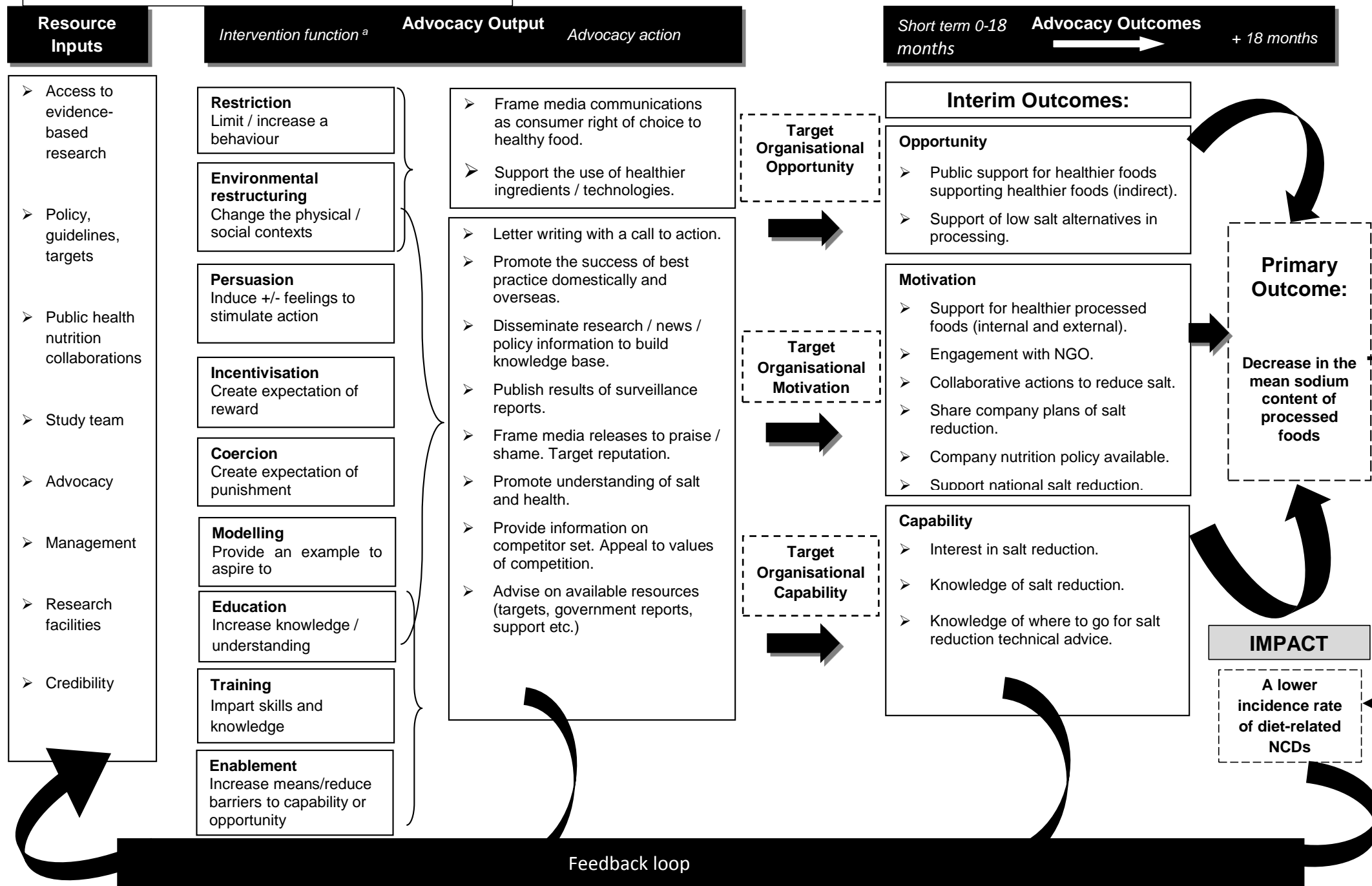

Supplement: Supplementary file 1 — Intervention progam logic model. [file 12889_2016_2743_MOESM1_ESM.pdf]
