# Supplementary material for: Protocol for a cluster-randomised trial to determine the effects of advocacy actions on the salt content of processed foods
Source: BMC Public Health. 2016 Jan 25;16:75. doi: 10.1186/s12889-016-2743-4 (PMC4727283; doi:10.1186/s12889-016-2743-4)
Supplement: Supplementary file 2 — Interim outcomes: examples of the types and sources of data, and measures. Description of data: description of the interim measures, examples of the types of data to be collected, the data sources, and the measure. (PDF 175 kb) [file 12889_2016_2743_MOESM2_ESM.pdf]

**Additional File 2** Interim outcomes: examples of the types and sources of data and measures.

| Interim outcome | Examples of data types | Examples of data sources | Measures |
|---|---|---|---|
| The number of and type of publicly-available statements from food companies expressing support / non-support for healthier processed foods. | Company media releases<br><br>Company statements<br><br>Company news / comments featured in electronic press | Internet; company websites, trade and public health newsletters | Count of statements / media items |
| The number of food companies with a nutrition policy published on their website. | Nutrition policy<br><br>Nutrition statement | Company website | Count of policies / statements |
| The level of engagement with the non-government organisation, as measured by a count of pre-defined methods of communication from the food company. | Email, formal meetings, presentations | Advocacy activity logs | Count of communications by type |
| The number of companies supporting the use of salt replacers / technologies in food processing to reduce the quantity of sodium required in processing. | Survey questionnaire | Self-administered survey questionnaire | Continuous and categorical measures relating to knowledge, attitudes and behaviours re. salt replacers |
| The number of companies supporting national salt reduction initiatives. | Public support for the Food and Health Dialogue | Food and Health Dialogue website | Count of companies and commitments |
| The number of companies providing evidence of planned salt reduction | Shared plans / commitments to salt reduction | Self-administered survey questionnaire<br><br>Supporting statements. Public commitment | Count of plans / commitments |
